# Outcomes and Risk Factors Associated With SARS-CoV-2 Infection in a North American Registry of Patients With Multiple Sclerosis

**DOI:** 10.1001/jamaneurol.2021.0688

**Published:** 2021-03-19

**Authors:** Amber Salter, Robert J. Fox, Scott D. Newsome, June Halper, David K. B. Li, Pamela Kanellis, Kathleen Costello, Bruce Bebo, Kottil Rammohan, Gary R. Cutter, Anne H. Cross

**Affiliations:** 1 Washington University School of Medicine in St Louis, St Louis, Missouri; 2 Cleveland Clinic, Cleveland, Ohio; 3 Johns Hopkins University School of Medicine, Baltimore, Maryland; 4 Consortium of MS Centers, Hackensack, New Jersey; 5 The University of British Columbia, Vancouver, British Columbia, Canada; 6 MS Society of Canada, Toronto, Ontario, Canada; 7 National Multiple Sclerosis Society, Chicago, Illinois; 8 University of Miami School of Medicine, Miami, Florida; 9 The University of Alabama at Birmingham, Birmingham

## Abstract

**Importance:**

Emergence of SARS-CoV-2 causing COVID-19 prompted the need to gather information on clinical outcomes and risk factors associated with morbidity and mortality in patients with multiple sclerosis (MS) and concomitant SARS-CoV-2 infections.

**Objective:**

To examine outcomes and risk factors associated with COVID-19 clinical severity in a large, diverse cohort of North American patients with MS.

**Design, Setting, and Participants:**

This analysis used deidentified, cross-sectional data on patients with MS and SARS-CoV-2 infection reported by health care professionals in North American academic and community practices between April 1, 2020, and December 12, 2020, in the COVID-19 Infections in MS Registry. Health care professionals were asked to report patients after a minimum of 7 days from initial symptom onset and after sufficient time had passed to observe the COVID-19 disease course through resolution of acute illness or death. Data collection began April 1, 2020, and is ongoing.

**Exposures:**

Laboratory-positive SARS-CoV-2 infection or highly suspected COVID-19.

**Main Outcomes and Measures:**

Clinical outcome with 4 levels of increasing severity: not hospitalized, hospitalization only, admission to the intensive care unit and/or required ventilator support, and death.

**Results:**

Of 1626 patients, most had laboratory-positive SARS-CoV-2 infection (1345 [82.7%]), were female (1202 [74.0%]), and had relapsing-remitting MS (1255 [80.4%]). A total of 996 patients (61.5%) were non-Hispanic White, 337 (20.8%) were Black, and 190 (11.7%) were Hispanic/Latinx. The mean (SD) age was 47.7 (13.2) years, and 797 (49.5%) had 1 or more comorbidity. The overall mortality rate was 3.3% (95% CI, 2.5%-4.3%). Ambulatory disability and older age were each independently associated with increased odds of all clinical severity levels compared with those not hospitalized after adjusting for other risk factors (nonambulatory: hospitalization only, odds ratio [OR], 2.8 [95% CI, 1.6-4.8]; intensive care unit/required ventilator support, OR, 3.5 [95% CI, 1.6-7.8]; death, OR, 25.4 [95% CI, 9.3-69.1]; age [every 10 years]: hospitalization only, OR, 1.3 [95% CI, 1.1-1.6]; intensive care unit/required ventilator support, OR, 1.3 [95% CI, 0.99-1.7]; death, OR, 1.8 [95% CI, 1.2-2.6]).

**Conclusions and Relevance:**

In this registry-based cross-sectional study, increased disability was independently associated with worse clinical severity including death from COVID-19. Other risk factors for worse outcomes included older age, Black race, cardiovascular comorbidities, and recent treatment with corticosteroids. Knowledge of these risk factors may improve the treatment of patients with MS and COVID-19 by helping clinicians identify patients requiring more intense monitoring or COVID-19 treatment.

## Key Points

**Question:**

How do patients with multiple sclerosis (MS) who have COVID-19 fare and are there patient and disease characteristics associated with worse outcome?

**Findings:**

In this registry-based cross-sectional study of 1626 North American patients with MS and COVID-19 infection, ambulatory disability, both nonambulatory and requiring assistance to walk, was independently associated with increased odds of poor clinical severity levels after adjusting for other risk factors. Other factors including older age, male sex, Black race, cardiovascular comorbidities, and corticosteroid use in the past 2 months were associated with increased odds of increasing clinical severity compared with those not requiring hospitalization or worse.

**Meaning:**

Identification of risk factors can improve the treatment of patients with MS and COVID-19 by alerting clinicians of patients requiring more intense treatment or monitoring.

## Teaser

This cross-sectional study examines outcomes and risk factors associated with COVID-19 clinical severity in a large, diverse cohort of North American patients with multiple sclerosis.

## Introduction

At the beginning of the COVID-19 pandemic caused by SARS-CoV-2, many concerns regarding the risk of infection and its consequences were raised, spurring global efforts to gather information.^1,2^ Multiple sclerosis (MS) is a common central nervous system disease, with estimated prevalence of 914 000 in the US and 90 000 in Canada.^3,4^ Because MS involves the immune system and MS disease-modifying therapies (DMTs) alter the immune response, special concerns existed regarding the severity of COVID-19 in patients with MS and the possible effects of MS DMTs on COVID-19 outcomes.

Risk factors for poorer outcomes that have been identified in the general population include older age, male sex, having specific comorbid conditions, and select race/ethnicity.^5,6,7^ Early reports with considerably smaller sample sizes indicated that in patients with MS, age and disability were associated with a more severe course of COVID-19.^1,8,9^ Larger definitive studies have been needed to further understand the effect of comorbidities and patient characteristics.

The COVID-19 Infections in MS (COViMS) Registry is a North American registry set up early in the pandemic for health care professionals to report cases of SARS-CoV-2 infection in people with MS and related conditions. Its goal is to understand how people with MS fare on exposure to the virus and to identify factors that might be associated with COVID-19 outcomes. To enable ease of information sharing, COViMS Registry data collection was harmonized with the Global Data Sharing initiative acquiring information on SARS-CoV-2 infection worldwide. While similar strategies were used across the Global Data Sharing initiative, North American–specific variations in SARS-CoV-2 infections merit examination.^10^ Here, using data from the COViMS Registry, we investigated factors associated with severe outcomes in a large and diverse population of North Americans with MS and SARS-CoV-2 infection.

## Methods

The COViMS Registry collects data from health care professionals in North America and is jointly supported by the Consortium of Multiple Sclerosis Centers, National MS Society (US), and MS Society of Canada. Broad outreach using a variety of mediums to inform the neurologic community was conducted by the sponsoring organizations to enhance robustness and representativeness of data collected.

### Study Design

Patients with MS and laboratory-positive or highly suspected infection with SARS-CoV-2 were eligible for inclusion in the COViMS Registry. Health care professionals were asked to report patients after a minimum of 7 days from initial infectious symptom onset and when sufficient time had passed to observe the COVID-19 disease course through resolution of acute illness or death. COVID-19 diagnosis in laboratory-confirmed patients was based on either polymerase chain reaction or serology tests. The data collection instrument was developed by a group of MS clinicians and epidemiologists and harmonized with the Global Data Sharing initiative COVID-19 core data set^11^ when possible. The instrument was designed to answer the main questions but to be brief to minimize the time to enter data during this crisis. Starting April 1, 2020, deidentified, cross-sectional patient-level data were entered into a secure, Health Insurance Portability and Accountability Act–compliant web-based REDCap^12^ database housed at Washington University in St Louis. Research participant protection was sought from the Washington University in St Louis institutional review board for housing COViMS Registry data, who determined it to be not human subjects research and therefore exempt from active institutional review board oversight at Washington University in St Louis and did not require patient consent. Individual contributors were encouraged to confer with their local institutional review board regarding requirements. Data use agreements govern the use of some data that were contributed, and thus access to individual-level data are not permitted by outside researchers. This study followed the Strengthening the Reporting of Observational Studies in Epidemiology (STROBE) reporting guideline.

### Risk Factors

Demographic risk factors collected were sex (male, female, nonbinary), age, race (White; Black or African American [Black]; Asian; American Indian, Alaska Native, or Indigenous Canadian; Native Hawaiian or other Pacific Islander; other; or unknown), ethnicity (Hispanic or Latinx, not Hispanic or Latinx, other, or unknown), and country and state/province at time of infection.

MS disease characteristics collected include MS disease duration and ambulation milestones (fully ambulatory, walks with assistance, nonambulatory). Comorbid conditions were selected from cancer, cardiovascular disease, cerebrovascular disease, chronic kidney disease, chronic liver disease, chronic lung disease, chronic neurological and/or neuromuscular disease, diabetes, hypertension, immunodeficiency disease, morbid obesity, and other. Comorbid conditions were also summed to categorize the count as 0 (reference), 1, 2, and 3 or more comorbidities. Cigarette use history was captured as never, past, current, or unknown. Glucocorticoid treatment during the prior 2 months was ascertained. Current DMT at the time of SARS-CoV-2 infection was reported as one of the following: alemtuzumab, azathioprine, cladribine, daclizumab, dimethyl fumarate, diroximel fumarate, fingolimod, glatiramer acetate, hematopoietic stem cell transplant, interferon-beta, intravenous immunoglobulin, methotrexate, mitoxantrone, mycophenolate, natalizumab, ocrelizumab, ofatumumab, ozanimod, rituximab, siponimod, teriflunomide, other, none, and unknown.

### Outcome

Clinicians summarized events of the COVID-19 course by indicating whether the patient was hospitalized, admitted to the intensive care unit (ICU), required ventilator support, or died, with response options of yes, no, or unknown to each. Responses of unknown were considered as not having the outcome. These events were used to create a single outcome of severe COVID-19 with 4 levels of increasing severity: not hospitalized, hospitalization only, ICU and/or required ventilator support, and death. If a patient had more than 1 of the events, they were assigned to the highest level of event that occurred.^13^

### Statistical Analysis

Cohort characteristics were summarized using mean (SD) for continuous variables, median (interquartile range) for ordinal variables, and frequencies (%) for categorical variables. Comparisons between groups were made using *t* tests and χ^2^ or Fisher exact tests, as appropriate. Age- and race-specific outcome proportions and 95% exact CIs were reported and differences evaluated using a χ^2^ test. A Cochran-Mantel-Haenszel was used to evaluate differences across age and race groups.

A multivariable multinomial logistic regression model was constructed to examine the assocations of risk factors with COVID-19 clinical severity with not hospitalized as the reference level. The multinomial model was used after detecting violations in the proportional odds assumption for an ordinal logistic regression. The multivariable models included a fixed set of covariates including age (continuous), sex (reference, female), race (reference, White), ambulation (reference, fully ambulatory), cigarette smoking history (reference, never smoked), glucocorticoid use (reference, no), comorbidities, and DMTs. Comorbidities included were cardiovascular disease, chronic lung disease, diabetes, hypertension, and morbid obesity (reference, absence of comorbidity [no]); current DMTs were categorized as none (reference), interferons (interferon beta-1a, interferon beta-1b), glatiramer acetate, fumarates (dimethyl fumarate, diroximel fumarate), sphingosine 1-phosphate receptor modulators (fingolimod, siponimod, ozanimod), teriflunomide, ocrelizumab, natalizumab, rituximab, and other (all <10 patients reported taking the DMT: alemtuzumab, cladribine, intravenous immunoglobulin, azathioprine, methotrexate, mitoxantrone, mycophenolate, ofatumumab, other). Associations were reported using odds ratios and 95% CIs. Multicollinearity was assessed using the correlation matrix for the parameter estimates and factors with associations more than 0.8 removed. Sensitivity analyses were conducted to examine the associations with outcomes, including only laboratory-positive cases and the number of comorbid conditions using similar methodology. Statistical analyses were conducted using SAS statistical software version 9.4 (SAS Institute Inc). Two-sided *P *values were statistically significant at .05.

## Results

### Population

As of December 12, 2020, 1626 patients with MS were reported in the COViMS Registry by more than 150 North American academic and private practices in 47 US states, Puerto Rico, 4 Canadian provinces, and Mexico (eFigure in the Supplement). Demographic and clinical characteristics are shown in Table 1. Most patients were laboratory positive (1345 [82.7%]), were female (1202 [74.0%]), had relapsing-remitting MS (1255 [80.4%]), and were in the US at COVID-19 onset (1547 [97.0%]). The mean (SD) age was 47.7 (13.2) years and MS disease duration was 13.1 (9.9) years. Approximately half had 1 or more comorbidity. Hypertension (358 [22.0%]), morbid obesity (179 [11.0%]), and diabetes (148 [9.1%]) were most frequently reported.

**Table 1.** Demographic and Clinical Characteristics Overall and by Clinical Outcome Severity^a^

Characteristic	No. (%)					*P* value
	Overall (N = 1626)	Not hospitalized (n = 1293)	Hospitalization only (n = 200)	ICU and/or required ventilator support (n = 79)	Death (n = 54)	
Sex						
Female	1202 (74.0)	985 (76.3)	132 (66.0)	57 (72.2)	28 (51.9)	<.001^b^
Male	421 (25.9)	305 (23.6)	68 (34.0)	22 (27.8)	26 (48.1)	
Age, mean (SD), y	47.7 (13.2)	46.0 (12.7)	52.2 (12.9)	53.4 (12.5)	62.4 (11.9)	<.001^c^
Race/ethnicity						
Non-Hispanic White	996 (61.5)	802 (62.4)	121 (60.5)	38 (48.1)	35 (64.8)	.001^b^
Black or African American	337 (20.8)	240 (18.7)	56 (28.0)	27 (34.2)	14 (25.9)	
Hispanic or Latinx	190 (11.7)	161 (12.5)	16 (8.0)	11 (13.9)	2 (3.7)	
Other^d^/unknown	96 (5.9)	83 (6.5)	7 (3.5)	3 (3.8)	3 (5.6)	
Country at time of COVID-19 onset						
United States	1547 (97.0)	1224 (96.8)	193 (96.5)	76 (98.7)	54 (100.0)	.68^e^
Canada	30 (1.9)	27 (2.1)	3 (1.5)	0	0	
Mexico	2 (0.13)	2 (0.16)	0	0	0	
Other	16 (1.0)	11 (0.87)	4 (2.0)	1 (1.3)	0	
US census region						
Northeast	365 (23.7)	272 (22.3)	58 (30.1)	13 (17.6)	22 (40.7)	.001^b^
Midwest	367 (23.8)	298 (24.4)	36 (18.7)	22 (29.7)	11 (20.4)	
South	671 (43.6)	552 (45.3)	74 (38.3)	29 (39.2)	16 (29.6)	
West	137 (8.9)	97 (8.0)	25 (13.0)	10 (13.5)	5 (9.3)	
Type of COVID-19 diagnosis						
Laboratory positive	1345 (82.7)	1035 (77.0)	186 (13.8)	75 (5.6)	49 (3.6)	<.001^b^
Suspected COVID-19, not confirmed	281 (17.3)	258 (91.8)	14 (5.0)	4 (1.4)	5 (1.8)	
Disease duration, mean (SD), y	13.1 (9.9)	12.2 (9.5)	15.1 (10.7)	16.9 (10.6)	21.3 (10.6)	<.001^c^
MS clinical course						
RRMS/CIS	1275 (82.0)	1091 (87.6)	130 (66.7)	37 (54.4)	17 (36.2)	<.001^b^
Progressive MS	280 (18.0)	154 (12.4)	65 (33.3)	31 (45.6)	30 (63.8)	
Ambulatory status						
Fully ambulatory	1184 (75.2)	1033 (82.0)	107 (54.3)	36 (50.7)	8 (17.0)	<.001^b^
Walk with assistance	241 (15.3)	154 (12.2)	57 (28.9)	21 (29.6)	9 (19.1)	
Nonambulatory	149 (9.5)	72 (5.7)	33 (16.8)	14 (19.7)	30 (63.8)	
DMT at time of COVID-19						
Alemtuzumab	9 (0.57)	6 (0.48)	1 (0.51)	1 (1.4)	1 (2.1)	<.001^b^
Cladribine	14 (0.89)	13 (1.0)	1 (0.51)	0	0	
Dimethyl fumarate	208 (13.3)	179 (14.3)	22 (11.1)	4 (5.6)	3 (6.3)	
Diroximel fumarate	3 (0.19)	3 (0.24)	0	0	0	
Fingolimod	106 (6.8)	97 (7.8)	3 (1.5)	6 (8.3)	0	
Glatiramer acetate	84 (5.4)	70 (5.6)	12 (6.1)	0	2 (4.2)	
Interferon beta	53 (3.4)	49 (3.9)	2 (1.0)	1 (1.4)	1 (2.1)	
IVIG	6 (0.38)	6 (0.48)	0	0	0	
Methotrexate	2 (0.13)	2 (0.16)	0	0	0	
Mycophenolate	2 (0.13)	1 (0.08)	0	0	1 (2.1)	
Natalizumab	170 (10.8)	154 (12.3)	12 (6.1)	1 (1.4)	3 (6.3)	
Ocrelizumab	484 (30.9)	367 (29.4)	77 (38.9)	29 (40.3)	11 (22.9)	
Ofatumumab	3 (0.19)	2 (0.16)	1 (0.51)	0	0	
Ozanimod	1 (0.06)	1 (0.08)	0	0	0	
Rituximab	77 (4.9)	48 (3.8)	19 (9.6)	7 (9.7)	3 (6.3)	
Siponimod	17 (1.1)	11 (0.88)	4 (2.0)	0	2 (4.2)	
Teriflunomide	82 (5.2)	69 (5.5)	8 (4.0)	2 (2.8)	3 (6.3)	
Other	10 (0.64)	4 (0.32)	4 (2.0)	1 (1.4)	1 (2.1)	
None	237 (15.1)	168 (13.4)	32 (16.2)	20 (27.8)	17 (35.4)	
Glucocorticoid during the last 2 mo						
No	1333 (89.0)	1069 (90.1)	165 (85.1)	65 (91.5)	34 (75.6)	<.001^b^
Yes	63 (4.2)	37 (3.1)	18 (9.3)	4 (5.6)	4 (8.9)	
Unknown	101 (6.7)	81 (6.8)	11 (5.7)	2 (2.8)	7 (15.6)	
Comorbidities						
No	769 (47.7)	664 (51.9)	72 (36.2)	28 (35.9)	5 (9.3)	<.001^b^
Yes	797 (49.5)	577 (45.1)	125 (62.8)	49 (62.8)	46 (85.2)	
Unknown	45 (2.8)	39 (3.0)	2 (1.0)	1 (1.3)	3 (5.6)	
Cancer	62 (3.8)	47 (3.6)	10 (5.0)	1 (1.3)	4 (7.4)	.24^b^
Cardiovascular disease	92 (5.7)	45 (3.5)	22 (11.0)	10 (12.7)	15 (27.8)	<.001^b^
Cerebrovascular disease	25 (1.5)	10 (0.77)	11 (5.5)	4 (5.1)	0	<.001^b^
Chronic kidney disease	19 (1.2)	9 (0.70)	2 (1.0)	2 (2.5)	6 (11.1)	<.001^b^
Chronic liver disease	12 (0.74)	8 (0.62)	0	1 (1.3)	3 (5.6)	<.001^b^
Chronic lung disease	116 (7.1)	78 (6.0)	20 (10.0)	13 (16.5)	5 (9.3)	.001^b^
Chronic neurological disease	79 (4.9)	51 (3.9)	14 (7.0)	8 (10.1)	6 (11.1)	.003^b^
Diabetes	148 (9.1)	83 (6.4)	39 (19.5)	15 (19.0)	11 (20.4)	<.001^b^
Hypertension	358 (22.0)	244 (18.9)	60 (30.0)	26 (32.9)	28 (51.9)	<.001^b^
Immunodeficiency disease	34 (2.1)	21 (1.6)	6 (3.0)	3 (3.8)	4 (7.4)	.01^b^
Morbid obesity	179 (11.0)	122 (9.4)	31 (15.5)	17 (21.5)	9 (16.7)	<.001^b^
Other	245 (15.1)	185 (14.3)	28 (14.0)	9 (11.4)	23 (42.6)	<.001^b^
Comorbidity count						
0	833 (51.2)	720 (55.7)	75 (37.5)	30 (38.0)	8 (14.8)	<.001^b^
1	422 (26.0)	341 (26.4)	54 (27.0)	15 (19.0)	12 (22.2)	
2	229 (14.1)	159 (12.3)	43 (21.5)	14 (17.7)	13 (24.1)	
≥3	142 (8.7)	73 (5.6)	28 (14.0)	20 (25.3)	21 (38.9)	

Abbreviations: CIS, clinically isolated syndrome; DMT, disease-modifying therapy; ICU, intensive care unit; IVIG, intravenous immunoglobulin; MS, multiple sclerosis; RRMS, relapsing-remitting multiple sclerosis.

^a^ Data not available for all individuals. Missing values: sex, 2; age, 16; race, 7; country at time of COVID-19 onset, 31; census region, 7; disease duration, 96; MS clinical course, 65; ambulatory status, 52; glucocorticoid during the last 2 months, 129; DMT at time of COVID-19, 58; have comorbidities, 15.

^b^ Pearson χ^2^ test was used to determine the *P *value.

^c^ Analysis of variance was used to determine the *P *value.

^d^ Other races include Asian; American Indian, Alaska Native, or Indigenous Canadian; and Native Hawaiian or other Pacific Islander.

^e^ Fisher exact test was used to determine the *P *value.

### COVID-19 Symptoms

**Figure 1.**
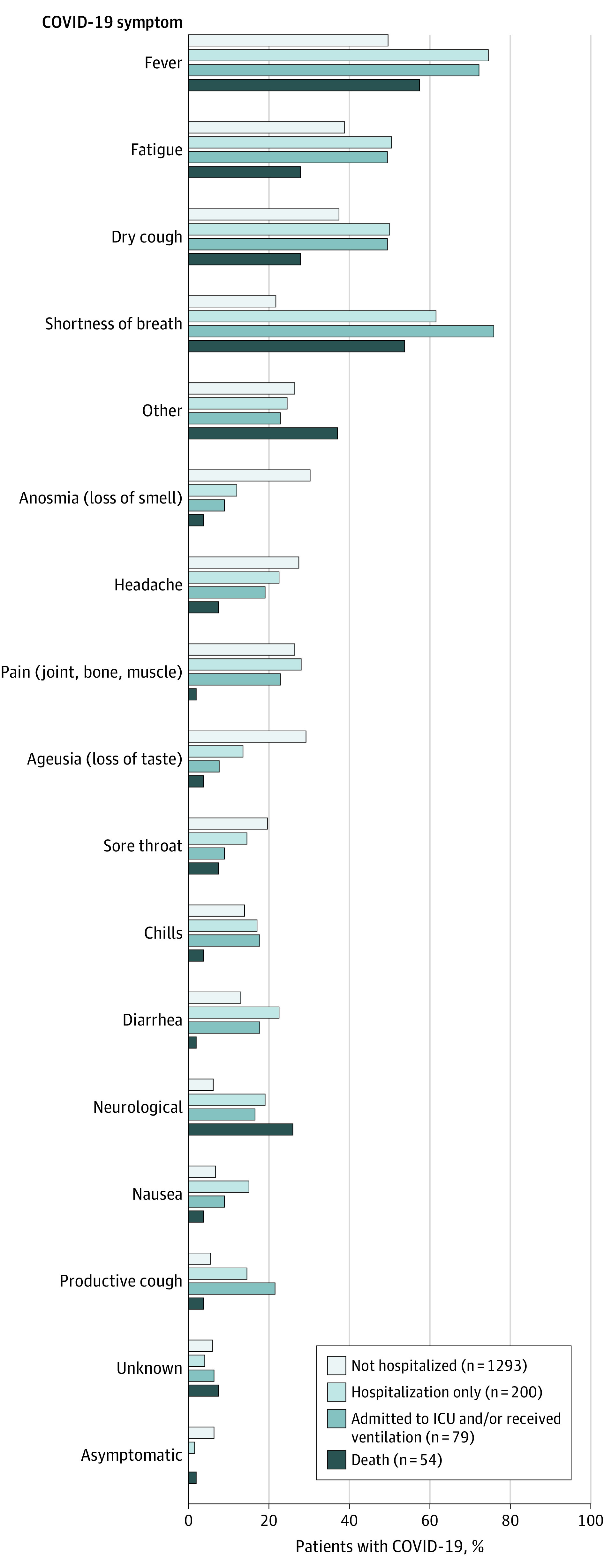
Frequency of COVID-19 Symptoms for Each COVID-19 Clinical Severity Level ICU indicates intensive care unit.

Fewer than 55% (n = 878) reported fever as a symptom of COVID-19. Dry cough and fatigue were each reported in about 40% of patients (637 [39.2%] and 657 [40.4%], respectively), with shortness of breath in 30.3% (n = 492). Anosmia, ageusia, pain, and headache were each reported in approximately 25% of patients (423 [26%], 412 [25.3%], 416 [25.6%], and 418 [25.7%], respectively) (Figure 1). Neurological symptoms were reported in 144 patients (8.9%). Of those with neurological symptoms, 64 (44.4%) reported motor dysfunction, and 37 (25.7%) reported cognitive dysfunction. Eighty-five cases (5.2%) were reported as asymptomatic. Symptoms lasted for 14 or more days in more than half of patients (0-6 days, 214 [20.8%]; 7-13 days, 285 [27.7%]; 14-20 days, 284 [27.6%]; and ≥21 days, 247 [24.0%]).

### COVID-19 Outcomes

**Figure 2.**
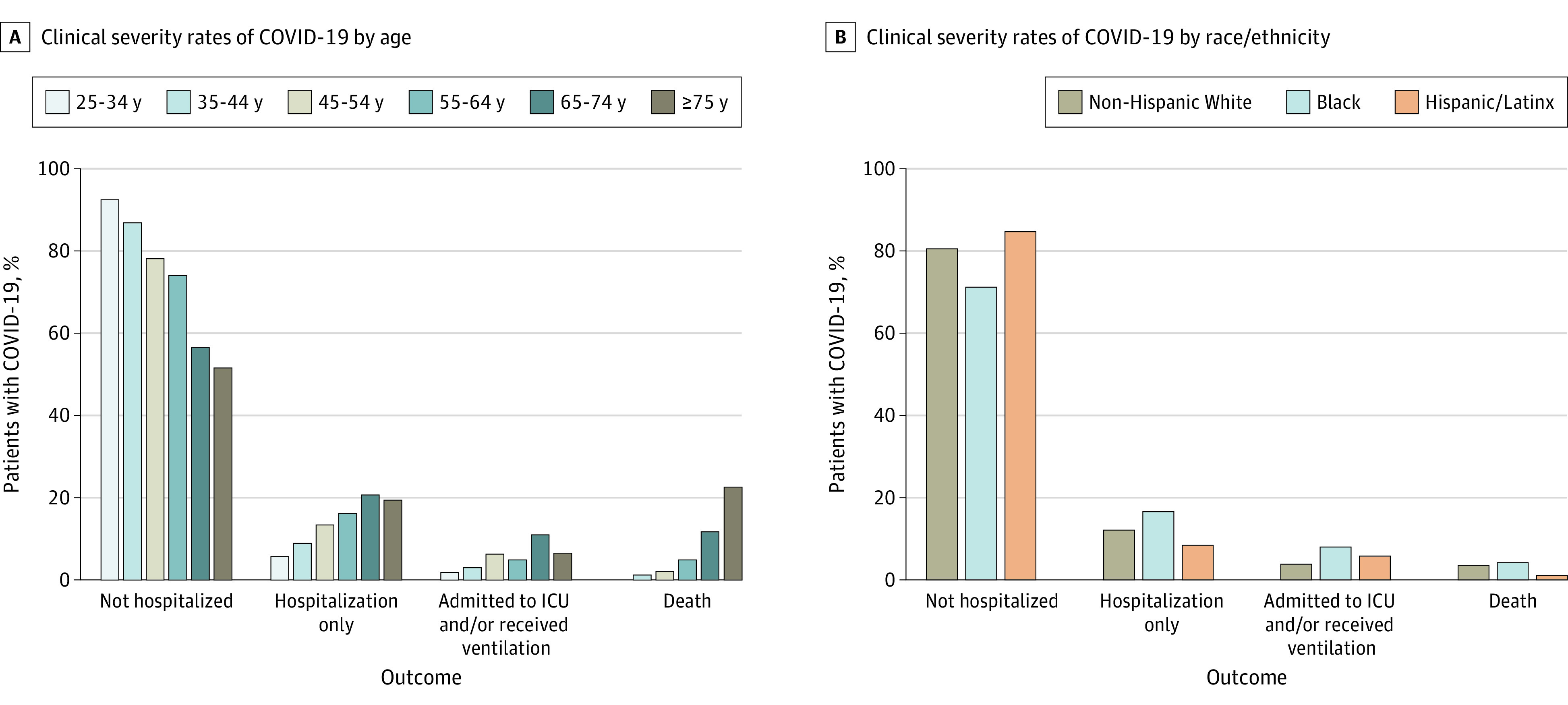
COVID-19 Clinical Severity Rates by Age and Race/Ethnicity ICU indicates intensive care unit.

Regarding individual components of the outcome, 425 (26.1%) visited an emergency department, 320 (19.7%) were hospitalized, 112 (6.9%) had pneumonia, 104 (6.4%) were admitted to the ICU, and 61 (3.8%) required ventilator support. Overall mortality rate was 3.3% (n = 54; 95% CI, 2.5%-4.3%). Of those who died, 43 (79.6%) were hospitalized, 29 (53.7%) were admitted to the ICU, and 25 (46.3%) required ventilator support. Mortality increased with age (Figure 2A), with no deaths in those younger than 35 years. The mortality rate was 1.2% (95% CI, 0.4%-2.9%) for individuals aged 35 to 44 years, 2.1% (95% CI, 1.0%-4.0%) for those aged 45 to 54 years, 4.9% (95% CI, 2.8%-7.8%) for those aged 55 to 64 years, 11.7% (95% CI, 7.0%-18.1%) for those aged 65 to 74 years, and 22.6% (95% CI, 9.6%-41.1%) for those 75 years or older.

The mortality rate was 3.5% (95% CI, 2.5%-4.9%) for White patients with MS, 4.2% (95% CI, 2.3%-6.9%) for Black patients with MS, and 1.1% (95% CI, 0.1%-3.8%) for Hispanic/Latinx patients with MS. eTable 1 in the Supplement reports the age- and race-specific rates for each level of clinical severity. Higher proportions of younger-aged Black patients with MS had worse outcomes vs younger White patients (Figure 2B). Clinical severity in 190 Hispanic/Latinx patients with MS was generally similar to those of White patients with MS across age levels.

### Risk Factors

Compared with patients with MS who were not hospitalized, older age was associated with an increased risk of each level of COVID-19 severity after adjusting for covariates (Table 2). For every 10-year increase in age, a 30% increased risk of both hospitalization alone and ICU admission and/or ventilation was identified. Notably, there was a 76.5% increased risk of death for every 10-year age increase. Male sex was associated with 41% increased odds of hospitalization and more than 3-fold increased risk of death. Black patients with MS had a 47% increased odds of hospitalization alone, more than a 2-fold increased risk of ICU admission and/or ventilation, yet no increased risk of death. No associations with poorer clinical severity were observed in Hispanic/Latinx individuals and other races.

**Table 2.** Multivariable Multinomial Logistic Regression Model for the Clinical Severity Outcome

Risk factor	COVID-19 clinical course outcome level^a^					
	Hospitalization only, OR (95% CI)	*P* value	ICU and/or required ventilator support, OR (95% CI)	*P* value	Death, OR (95% CI)	*P* value
Age (every 10-y increase)	1.32 (1.12-1.56)	<.001	1.29 (0.99-1.67)	.06	1.77 (1.20-2.59)	.004
Sex (male vs female)	1.41 (0.98-2.03)	.06	1.00 (0.54-1.83)	.99	3.12 (1.46-6.65)	.003
Race/ethnicity						
Non-Hispanic White	1 [Reference]	NA^b^	1 [Reference]	NA^b^	1 [Reference]	NA^b^
Black	1.47 (0.98-2.22)	.03	2.28 (1.22-4.23)	.08	1.60 (0.65-3.93)	.60
Hispanic or Latinx	0.77 (0.41-1.44)	.34	1.76 (0.76-4.09)	.50	0.57 (0.10-3.18)	.23
Other^c^/unknown	0.82 (0.33-2.08)	.61	0.94 (0.20-4.36)	.50	2.95 (0.45-19.5)	.25
Ambulation						
Fully ambulatory	1 [Reference]	NA^b^	1 [Reference]	NA^b^	1 [Reference]	NA^b^
Walk with assistance	2.18 (1.42-3.34)	.23	2.35 (1.22-4.54)	.48	3.14 (0.99-9.95)	.32
Nonambulatory	2.82 (1.64-4.85)	.01	3.53 (1.59-7.81)	.02	25.4 (9.34-69.1)	<.001
Smoking status						
Never	1 [Reference]	NA^b^	1 [Reference]	NA^b^	1 [Reference]	NA^b^
Current	1.09 (0.52-2.29)	.99	NA^b^	.95	2.88 (0.67-12.4)	.24
Past	1.08 (0.73-1.60)	.95	1.10 (0.61-1.99)	.95	1.32 (0.59-2.94)	.76
Unknown	1.22 (0.58-2.57)	.71	1.46 (0.48-4.45)	.94	1.23 (0.26-5.94)	.77
Cardiovascular disease (yes vs no)	1.91 (1.02-3.59)	.04	1.46 (0.54-3.98)	.46	3.15 (1.18-8.45)	.02
Chronic lung disease (yes vs no)	1.29 (0.71-2.36)	.41	2.12 (0.97-4.67)	.06	1.10 (0.30-4.05)	.89
Diabetes (yes vs no)	2.46 (1.50-4.04)	<.001	1.85 (0.87-3.95)	.11	1.07 (0.39-2.93)	.90
Hypertension (yes vs no)	1.11 (0.73-1.69)	.63	1.08 (0.58-2.04)	.80	3.14 (1.38-7.15)	.006
Morbid obesity (yes vs no)	1.69 (1.03-2.75)	.04	2.87 (1.48-5.54)	.002	2.49 (0.92-6.75)	.07
Disease-modifying therapy						
None	1 [Reference]	NA^b^	1 [Reference]	NA^b^	1 [Reference]	NA^b^
Fumarates	0.99 (0.52-1.88)	.90	0.26 (0.08-0.82)	.98	0.40 (0.09-1.70)	.30
S1PR	0.65 (0.26-1.61)	.23	0.77 (0.28-2.14)	.94	0.86 (0.15-4.93)	.89
Glatiramer acetate	1.15 (0.51-2.61)	.73	NA^b^	.96	0.86 (0.16-4.56)	.89
Interferons	0.35 (0.08-1.57)	.11	0.29 (0.04-2.32)	.98	0.56 (0.06-5.49)	.75
Natalizumab	0.67 (0.31-1.45)	.18	0.09 (0.01-0.73)	.98	0.80 (0.19-3.44)	.96
Ocrelizumab	1.63 (0.98-2.72)	.009	0.91 (0.46-1.80)	.94	0.47 (0.17-1.30)	.25
Other	1.21 (0.45-3.24)	.70	0.50 (0.10-2.38)	.96	0.91 (0.18-4.73)	.83
Rituximab	4.56 (2.10-9.90)	<.001	1.92 (0.61-6.07)	.91	2.81 (0.45-17.70)	.11
Teriflunomide	0.83 (0.34-2.02)	.58	0.30 (0.06-1.37)	.98	0.48 (0.08-3.04)	.57
Glucocorticoid use in past 2 mo						
No	1 [Reference]	NA^b^	1 [Reference]	NA^b^	1 [Reference]	NA^b^
Unknown	0.94 (0.46-1.92)	.16	0.44 (0.10-1.94)	.19	2.13 (0.68-6.72)	.95
Yes	2.62 (1.33-5.17)	.009	1.57 (0.49-4.97)	.21	4.17 (1.13-15.4)	.13

Abbreviations: ICU, intensive care unit; NA, not applicable; OR, odds ratio; S1PR, sphingosine 1-phosphate receptor.

^a^ Reference level is not hospitalized.

^b^ Insufficient sample size to estimate OR.

^c^ Other races include Asian; American Indian, Alaska Native, or Indigenous Canadian; and Native Hawaiian or other Pacific Islander.

Requiring assistance to walk or being nonambulatory was independently associated with increased odds of all COVID-19 clinical severity levels after adjusting for covariates (Table 2). Requiring assistance to walk was associated with 2-fold or greater increased odds of all clinical severity levels. Being nonambulatory was associated with 2.8-fold increased odds of hospitalization alone and 3.5-fold increase for ICU admission and/or ventilation. A 25-fold increased odds of death for nonambulatory patients with MS compared with fully ambulatory patients was observed after adjustment for other risk factors. Cardiovascular disease was associated with a 91% increased risk of hospitalization alone and more than 3-fold increased odds of death from COVID-19. Hypertension was also associated with more than 3-fold increased risk of death but not other levels of clinical severity. Diabetes was associated with 2.5-fold increased odds of hospitalization but not other levels of clinical severity. Morbid obesity was associated with a 68.5% increased odds of hospitalization alone and an almost 3-fold increased odds of ICU admission and/or ventilation. No associations between COVID-19 clinical severity and cigarette smoking and chronic lung disease were observed.

Compared with those not taking any DMT, patients taking rituximab had a 4.5-fold increased odds of hospitalization for COVID-19; however, no other levels of clinical severity were associated with taking rituximab. Ocrelizumab use slightly increased the odds of hospitalization alone (odds ratio, 1.63). Fumarates and natalizumab treatments were each associated with decreased odds of ICU admission and/or ventilation. Notably, glucocorticoid use in the prior 2 months conferred approximately 2-fold increased risk of hospitalization and 4-fold increased risk of death.

### Sensitivity Analyses

SARS-CoV-2–laboratory positive patients were older, had a higher proportion of Black patients with MS, and more cardiovascular comorbidities compared with patients with suspected COVID-19 (eTable 2 in the Supplement). Analyses examining only the laboratory-positive patients showed risk factors that were consistent with the entire cohort at all clinical severity levels (eTable 3 in the Supplement). Results for the model including number of comorbidities were also consistent. Having 2 or more comorbid conditions was associated with increased odds of poorer clinical outcome compared with having no comorbidities (eTable 4 in the Supplement).

## Discussion

The COViMS Registry collected clinician-reported information on COVID-19 outcomes and risk factors from a large, diverse set of patients with MS in North America. SARS-CoV-2 infection was laboratory confirmed in more than 82% of reported cases. Increased neurologic disability was consistently associated with a large increased risk of severe clinical outcome after accounting for other risk factors. Although not all statistically significant, rituximab showed notable and consistent associations with worse outcomes compared with other DMTs. Older age, obesity, and several cardiovascular comorbidities were associated with more severe COVID-19 as well.

The COViMS Registry is uniquely positioned to explore the association of race and ethnicity with COVID-19 outcomes, which is of special interest in the diverse North American population. Compared with White race, Black race was associated with increased odds of ICU admission and/or ventilation and a nominally increased odds of hospitalization alone. However, no association of Black race with death from COVID-19 was observed in the COViMS Registry. Similarly, some studies in the general US population have not found increased mortality in Black patients with COVID-19 after adjustment for other risk factors.^14,15,16^ We did not collect measures of socioeconomic or essential worker status, which might have contributed toward explaining racial and age differences. Yet, an unmeasured confounder such as occupation or living conditions would need to impart a strong association, with an odds ratio of 2.4 or more, between race and COVID-19 clinical outcome to account for the worse outcomes observed for Black patients with MS compared with White patients with MS.^17^ Altogether, the worse outcomes observed for Black patients with MS indicate that close clinical monitoring of COVID-19 in these patients is warranted.

Compared with those not taking any DMT, rituximab use was associated with an increased risk of hospitalization. Although the risk for hospitalization for ocrelizumab was nominally increased, the association was not as strong as with rituximab. Differences in associations with COVID-19 outcomes for the 2 anti-CD20 monoclonal antibody therapies (rituximab and ocrelizumab) could be due to longer treatment duration with rituximab because ocrelizumab was more recently available. The Italian MS and COVID-19 (MUSC-19) registry did not distinguish between anti-CD20 therapies but showed worse clinical outcomes and higher risk with longer duration of anti-CD20 exposure.^18^ The COVID-19 Global Rheumatology Alliance registry found rituximab to be associated with 4-fold increased odds of death in people with rheumatologic disease compared with methotrexate-treated patients.^19^ While the French Covisep MS registry reported no association of anti-CD20 therapies with worse COVID-19 outcomes, its sample size of 347 may have limited the ability to detect associations.^9^ Taken together, these studies suggest increased risks of COVID-19 in people treated with rituximab.

The percent of COViMS Registry patients taking interferon beta and glatiramer acetate were 3.4% and 5.4%, respectively. Interestingly, the 2020 US market shares for interferon beta (14.5%) and glatiramer acetate (18.9%) are much larger than their proportions reported in the COViMS Registry. This may reflect prescribing patterns of a nonrandom sample of clinician reporters or might indicate a true effect of these DMTs on susceptibility to COVID-19. Notably, early data suggest a potential benefit for taking interferon beta on exposure to SARS-CoV-2^20^; currently, trials are underway to study the efficacy of interferon beta in COVID-19.^21,22^ In this study, fumarates and natalizumab were each associated with reduced risk of ICU admission and/or ventilation compared with those not receiving therapy. The reason for this is unknown but may be associated with the relative anti-inflammatory vs immunosuppressive effects of these medications.

Similar to MUSC-19^18^ and data reported in rheumatoid arthritis studies, we found an association of recent glucocorticoid use with increased risk of hospitalization and mortality.^23,24^ This was not completely unexpected, as glucocorticoids affect the immune system, reducing responsiveness to infections. Glucocorticoids sometimes are prescribed to treat the inflammatory cytokine storm of COVID-19, so the timing of glucocorticoid administration and type of glucocorticoid could play a role in COVID-19 outcomes.

With 54 deaths, we report the highest number of deaths thus far in a registry of patients with MS, to our knowledge. All deaths occurred in US patients. The COViMS Registry mortality rate of 3.3% was in line with the mortality rate reported by the Covisep MS registry (3.5%)^9^ but higher than the MUSC-19 cohort (1.5%).^18^ The 3.3% mortality rate in the COViMS Registry was also higher than the US mortality rate of 1.8% as of December 12, 2020.^25^ This may merely reflect greater tendency to report more severe cases in the voluntary COViMS Registry; however, numerous societal and public health issues may have also contributed. Risk factors for mortality identified in the COViMS Registry were older age, male sex, and hypertension, consistent with risk factors observed in the general population.^26^

No clear association of MS diagnosis with risk of developing COVID-19 could be established in this study because of the unknown numbers at risk in the MS populations from whom cases were reported. However, ambulatory disability from MS was strongly associated with worse COVID-19 outcomes, consistent with other studies.^8,9,23,27^ We did not capture information on assocations of SARS-CoV-2 infection within MS itself. Although some neurologic symptoms were noted in association with COVID-19, these symptoms were not prominent. Neurological symptoms have been reported in otherwise healthy persons with COVID-19.^28,29^

### Limitations

Limitations of this analysis include that reporting was voluntary by health care professionals, which may have biased reporting toward more severe cases. This would overestimate overall clinical severity in the COViMS Registry but have less effect on the comparisons among risk factors. Although cases were reported by more than 150 different sites spanning both academic and community practices across North America, a large proportion of COViMS Registry data, including 21 of 54 deaths, derived from the Northeast US. This was not unexpected given the disproportionate effect of COVID-19 on that region early in the pandemic. The COViMS Registry is ongoing, and regional shifts are expected as the pandemic expands within North America. Some patients may have behaved more cautiously and adhered more strictly to public health recommendations because of MS, but this was not captured.

## Conclusions

With more than 1600 reported patients with MS, the COViMS Registry provides evidence that ambulation disability, older age, and Black race are associated with worse COVID-19 clinical course in a North American MS population. Rituximab, recent treatment with corticosteroids, and risk factors known in the general population such as obesity and cardiovascular comorbidities were associated with worse COVID-19 clinical severity. Knowledge of these risk factors may enable clinicians caring for patients with MS to improve monitoring and treatment of COVID-19.

## Notes

Supplement. eTable 1. Age- and race-specific outcome rates eTable 2. Demographic and Clinical Characteristics By Diagnostic Method eTable 3. Multivariable multinomial logistic regression for COVID-19 laboratory positive MS patients only eTable 4. Multivariable multinomial logistic regression using the number of comorbid conditions eFigure. North American distribution of MS patients reported in COViMS Registry, A) Canada, B) United States
