# Supplementary material for: Outcomes and Risk Factors Associated With SARS-CoV-2 Infection in a North American Registry of Patients With Multiple Sclerosis
Source: JAMA Neurol. 2021 Mar 19;78(6):1–10. doi: 10.1001/jamaneurol.2021.0688 (PMC7980147; doi:10.1001/jamaneurol.2021.0688)
Supplement: Supplement. — eTable 1. Age- and race-specific outcome rates eTable 2. Demographic and Clinical Characteristics By Diagnostic Method eTable 3. Multivariable multinomial logistic regression for COVID-19 laboratory positive MS patients only eTable 4. Multivariable multinomial logistic regression using the number of comorbid conditions eFigure. North American distribution of MS patients reported in COViMS Registry, A) Canada, B) United States [file jamaneurol-e210688-s001.pdf]

Supplemental Online Content

Salter A, Fox RJ, Newsome SD, et al. Outcomes and risk factors associated with SARS-CoV-2 infection in a North American registry of patients with multiple sclerosis. *JAMA Neurol*. Published online March 19, 2021. doi:10.1001/jamaneurol.2021.0688

eTable 1. Age- and race-specific outcome rates

eTable 2. Demographic and Clinical Characteristics By Diagnostic Method

eTable 3. Multivariable multinomial logistic regression for COVID-19 laboratory positive MS patients only

eTable 4. Multivariable multinomial logistic regression using the number of comorbid conditions

eFigure. North American distribution of MS patients reported in COViMS Registry, A) Canada, B) United States

This supplemental material has been provided by the authors to give readers additional information about their work.

© 2021 Salter A et al. *JAMA Neurology.*

eTable 1. Age- and race-specific outcome rates.

Age Group	Race	Not hospitalized (N=1293)		Hospitalization only (N=200)		ICU and/or Ventilated (N=79)		Death (N=54)	
		Events/ Total	Percentage (95%CI)	Events/ Total	Percentage (95%CI)	Events/ Total	Percentage (95%CI)	Events/ Total	Percentage (95%CI)
15–24 years	Non- Hispanic White	12/13	92.3 (64.0, 99.8)	1/13	7.7 (0.2, 36.0)	0/13	-	0/13	-
	Black or African American	6/7	85.7 (42.1, 99.6)	0/7	-	1/7	14.3 (0.4, 57.9)	0/7	-
	Hispanic or Latinx	14/17	82.4 (56.6, 96.2)	2/17	11.8 (1.5, 36.4)	1/17	5.9 (0.1, 28.7)	0/17	-
	Other/Unknown	8/9	88.9 (51.8, 99.7)	1/9	11.1 (0.3, 48.2)	0/9	-	0/9	-
25–34 years	Non- Hispanic White	116/122	95.1 (89.6, 98.2)	5/122	4.1 (1.3, 9.3)	1/122	0.8 (0.0, 4.5)	0/122	-
	Black or African American	40/48	83.3 (69.8, 92.5)	6/48	12.5 (4.7, 25.2)	2/48	4.2 (0.5, 14.3)	0/48	-
	Hispanic or Latinx	34/36	94.4 (81.3, 99.3)	1/36	2.8 (0.1, 14.5)	1/36	2.8 (0.1, 14.5)	0/36	-
	Other/Unknown	20/21	95.2 (76.2, 99.9)	1/21	4.8 (0.1, 23.8)	0/21	-	0/21	-
35–44 years	Non- Hispanic White	211/233	90.6 (86.1, 94.0)	15/233	6.4 (3.6, 10.4)	5/233	2.1 (0.7, 4.9)	2/233	0.9 (0.1, 3.1)
	Black or African American	70/92	76.1 (66.1, 84.4)	13/92	14.1 (7.7, 23.0)	6/92	6.5 (2.4, 13.7)	3/92	3.3 (0.7, 9.2)
	Hispanic or Latinx	46/54	85.2 (72.9, 93.4)	7/54	13.0 (5.4, 24.9)	1/54	1.9 (0.0, 9.9)	0/54	-
	Other/Unknown	23/24	95.8 (78.9, 99.9)	1/24	4.2 (0.1, 21.1)	0/24	-	0/24	-
45–54 years	Non- Hispanic White	200/251	79.7 (74.2, 84.5)	36/251	14.3 (10.3, 19.3)	10/251	4.0 (1.9, 7.2)	5/251	2.0 (0.6, 4.6)
	Black or African American	64/92	69.6 (59.1, 78.7)	16/92	17.4 (10.3, 26.7)	10/92	10.9 (5.3, 19.1)	2/92	2.2 (0.3, 7.6)
	Hispanic or Latinx	43/53	81.1 (68.0, 90.6)	4/53	7.5 (2.1, 18.2)	5/53	9.4 (3.1, 20.7)	1/53	1.9 (0.0, 10.1)
	Other/Unknown	24/28	85.7 (67.3, 96.0)	1/28	3.6 (0.1, 18.3)	2/28	7.1 (0.9, 23.5)	1/28	3.6 (0.1, 18.3)

© 2021 Salter A et al. *JAMA Neurology.*

55–64 years	Non- Hispanic White	175/235	74.5 (68.4, 79.9)	38/235	16.2 (11.7, 21.5)	12/235	5.1 (2.7, 8.7)	10/235	4.3 (2.1, 7.7)
	Black or African American	47/64	73.4 (60.9, 83.7)	10/64	15.6 (7.8, 26.9)	2/64	3.1 (0.4, 10.8)	5/64	7.8 (2.6, 17.3)
	Hispanic or Latinx	15/19	78.9 (54.4, 93.9)	2/19	10.5 (1.3, 33.1)	2/19	10.5 (1.3, 33.1)	0/19	
	Other/Unknown	5/9	55.6 (21.2, 86.3)	3/9	33.3 (7.5, 70.1)	0/9	11.1 (0.3, 48.2)	1/9	
65–74 years	Non- Hispanic White	68/113	60.2 (50.5, 69.3)	24/113	21.2 (14.1, 29.9)	9/113	8.0 (3.7, 14.6)	12/113	10.6 (5.6, 17.8)
	Black or African American	9/23	39.1 (19.7, 61.5)	6/23	26.1 (10.2, 48.4)	5/23	21.7 (7.5, 43.7)	3/23	13.0 (2.8, 33.6)
	Hispanic or Latinx	4/6	66.7 (22.3, 95.7)	0/6	-	1/6	16.7 (0.4, 64.1)	1/6	16.7 (0.4, 64.1)
	Other/Unknown	1/3	33.3 (0.8, 90.6)	0/3	-	1/3	33.3 (0.8, 90.6)	1/3	33.3 (0.8, 90.6)
≥75 years	Non-Hispanic White	15/24	62.5 (40.6, 81.2)	2/24	8.3 (1.0, 27.0)	1/24	4.2 (0.1, 21.1)	6/24	25.0 (9.8, 46.7)
	Black or African American	0/6		4/6	66.7 (22.3, 95.7)	1/6	16.7 (0.4, 64.1)	1/6	16.7 (0.4, 64.1)
	Hispanic or Latinx	0/0	-	0/0	-	0/0	-	0/0	-
	Other/Unknown	1/1	100.0 (2.5, 100)	0/1	-	0/1	-	0/1	-
**Overall**	Non-Hispanic White	802/996	80.5 (77.9, 82.9)	121/996	12.1 (10.2, 14.3)	38/996	3.8 (2.7, 5.2)	35/996	3.5 (2.5, 4.9)
	Black or African American	240/337	71.2 (66.1, 76.0)	56/337	16.6 (12.8, 21.0)	27/337	8.0 (5.3, 11.4)	14/337	4.2 (2.3, 6.9)
	Hispanic or Latinx	161/190	84.7 (78.8, 89.5)	16/190	8.4 (4.9, 13.3)	11/190	5.8 (2.9, 10.1)	2/190	1.1 (0.1, 3.8)
	Other/Unknown	83/96	86.5 (78.0, 92.6)	7/96	7.3 (3.0, 14.4)	3/96	3.1 (0.6, 8.9)	3/96	3.1 (0.6, 8.9)

© 2021 Salter A et al. *JAMA Neurology.*

eTable 2. Demographic and Clinical Characteristics By Diagnostic Method.			
	**Laboratory Positive (N=1345)**	**Suspected COVID-19 (N=281)**	**p-value**
**Female**	988(73.6)	214(76.2)	0.61[c]
**Age** (years), mean (SD)	48.2(13.2)	45.1(12.8)	***<0.001[a]***
**Race**			***<0.001[c]***
Non-Hispanic White	831(62.1)	165(58.7)	
Black or African American	290(21.7)	47(16.7)	
Hispanic or Latinx	153(11.4)	37(13.2)	
Other/Unknown	64(4.8)	32(11.4)	
**Country at time of COVID-19 onset**			0.16[c]
United States	1277(96.7)	270(98.2)	
Canada	29(2.2)	1(0.36)	
Mexico	2(0.15)	0(0.0)	
Other	12(0.91)	4(1.5)	
**US Census region**			***<0.001[c]***
Northeast	300(23.6)	65(24.2)	
Midwest	354(27.9)	13(4.8)	
South	488(38.4)	183(68.0)	
West	129(10.1)	8(3.0)	
**Disease Duration** (years), mean (SD)	13.2(10.0)	12.6(9.5)	0.42[a]
**MS Clinical Course**			0.18[c]
RRMS/CIS	1041(81.4)	234(84.8)	
Progressive MS	238(18.6)	42(15.2)	
**Ambulatory Status**			0.092[c]
Fully ambulatory	964(74.4)	220(79.1)	
Walk with Assistance	200(15.4)	41(14.7)	
Non-ambulatory	132(10.2)	17(6.1)	
**DMT at Time of COVID-19**			***<0.001[c]***
Alemtuzumab	8(0.62)	1(0.36)	
Cladribine	12(0.93)	2(0.72)	
Dimethyl fumarate	177(13.7)	31(11.1)	
Diroximel fumarate	2(0.16)	1(0.36)	
Fingolimod	88(6.8)	18(6.5)	
Glatiramer acetate	76(5.9)	8(2.9)	
Interferon-beta	47(3.6)	6(2.2)	
IVIG	4(0.31)	2(0.72)	
Methotrexate	2(0.16)	0(0.0)	
Mycophenolate	2(0.16)	0(0.0)	

© 2021 Salter A et al. *JAMA Neurology.*

**eTable 2.** Demographic and Clinical Characteristics By Diagnostic Method.			
	**Laboratory Positive (N=1345)**	**Suspected COVID-19 (N=281)**	**p-value**
Natalizumab	144(11.2)	26(9.3)	
Ocrelizumab	400(31.0)	84(30.1)	
Ofatumumab	3(0.23)	0(0.0)	
Ozanimod	1(0.08)	0(0.0)	
Rituximab	46(3.6)	31(11.1)	
Siponimod	14(1.1)	3(1.1)	
Teriflunomide	71(5.5)	11(3.9)	
Other	7(0.54)	3(1.1)	
None	185(14.4)	52(18.6)	
**Glucocorticoid during the last 2 months?**			***<0.001[c]***
No	1138(90.4)	195(81.9)	
Yes	48(3.8)	15(6.3)	
Unknown	73(5.8)	28(11.8)	
**Have comorbidities?***			0.19[c]
No	651(48.8)	118(42.8)	
Yes	648(48.5)	149(54.0)	
Unknown	36(2.7)	9(3.3)	
**Cancer**	46(3.4)	16(5.7)	0.070[c]
**Cardiovascular disease**	87(6.5)	5(1.8)	***0.002[c]***
**Cerebrovascular disease**	24(1.8)	1(0.36)	0.077[c]
**Chronic kidney disease**	16(1.2)	3(1.1)	0.86[c]
**Chronic liver disease**	11(0.82)	1(0.36)	0.41[c]
**Chronic lung disease**	92(6.8)	24(8.5)	0.31[c]
**Chronic neurological disease**	68(5.1)	11(3.9)	0.42[c]
**Diabetes**	126(9.4)	22(7.8)	0.41[c]
**Hypertension**	309(23.0)	49(17.4)	***0.042[c]***
**Immunodeficiency disease**	30(2.2)	4(1.4)	0.39[c]
**Morbid obesity**	161(12.0)	18(6.4)	***0.007[c]***
**Other**	158(11.7)	87(31.0)	***<0.001[c]***

*Data not available for all subjects. Missing values: Gender = 2, Age = 16, Race = 7, Country at time of COVID-19 onset: = 31, Census region = 7, Disease Duration = 96, MS Clinical Course = 65, Ambulatory Status = 52, Glucocorticoid during the last 2 mo, DMT at Time of COVID-19 = 58, Have comorbidities? = 15
Values presented as Mean ± SD, Median [P25, P75] or N (column %).
p-values: a=ANOVA, b=Kruskal-Wallis test, c=Pearson's chi-square test, d=Fisher's Exact test.

© 2021 Salter A et al. *JAMA Neurology.*

eTable 3. Multivariable logistic regression for COVID-19 laboratory positive MS patients only.

Factor	Level	Hospitalization only			ICU and/or Ventilated			Death		
		OR	LCL	UCL	OR	LCL	UCL	OR	LCL	UCL
Age	(10 years)	1.358	1.143	1.613	1.355	1.038	1.769	2.149	1.412	3.27
Sex	Male vs Female	1.458	0.991	2.143	1.121	0.601	2.089	3.578	1.563	8.195
Race	Black/African American vs Non-Hispanic White	1.631	1.06	2.51	2.185	1.142	4.178	1.345	0.491	3.682
	Hispanic or Latinx vs Non- Hispanic White	0.766	0.393	1.493	1.727	0.707	4.218	0.621	0.101	3.816
	Other/Unknown vs Non- Hispanic White	1.08	0.383	3.044	0.679	0.083	5.535	2.819	0.283	28.121
Ambulation	Non-ambulatory vs Fully ambulatory	2.341	1.487	3.686	2.345	1.169	4.702	3.468	1.018	11.813
	Walk with Assistance vs Fully ambulatory	2.555	1.442	4.528	3.139	1.398	7.048	18.102	6.199	52.861
Smoking status	Current vs Never	0.972	0.437	2.164	-	-	-	1.584	0.25	10.02
	Past vs Never	0.996	0.65	1.526	1.096	0.59	2.037	1.363	0.587	3.16
	Unknown vs Never	1.25	0.583	2.681	1.58	0.512	4.882	1.7	0.318	9.09
Cardiovascular disease	Yes vs No	1.842	0.955	3.554	1.398	0.507	3.851	3.468	1.213	9.914
Chronic lung disease	Yes vs No	1.447	0.761	2.751	1.926	0.8	4.64	1.209	0.302	4.844
Diabetes	Yes vs No	2.523	1.469	4.334	1.893	0.842	4.259	1.008	0.339	2.993

© 2021 Salter A et al. *JAMA Neurology.*

Hypertension	Yes vs No	1.112	0.718	1.723		1.025	0.53	1.982		2.465	1.036	5.867
Morbid obesity	Yes vs No	1.485	0.885	2.49		2.435	1.205	4.923		3.354	1.177	9.553
Disease Modifying Therapy	Fumarates vs None	0.836	0.425	1.645		0.237	0.074	0.754		0.292	0.065	1.315
	S1PR vs None	0.499	0.187	1.328		0.61	0.202	1.845		0.655	0.106	4.052
	Glatiramer acetate vs None	0.921	0.392	2.162		-	-	-		0.304	0.034	2.74
	Injectables vs None	0.292	0.063	1.355		0.237	0.029	1.924		0.413	0.041	4.176
	Natalizumab vs None	0.545	0.243	1.222		0.081	0.01	0.637		0.643	0.144	2.863
	Ocrelizumab vs None	1.441	0.831	2.497		0.8	0.393	1.63		0.366	0.121	1.106
	Other vs None	0.866	0.295	2.539		0.437	0.09	2.129		0.637	0.116	3.487
	Rituximab vs None	4.885	1.984	12.028		2.718	0.808	9.142		1.205	0.087	16.621
	Teriflunomide vs None	0.827	0.332	2.061		0.267	0.057	1.259		0.41	0.057	2.946
Glucocorticoid use in the past 2 months	Unknown vs No	1.387	0.652	2.95		0.345	0.045	2.652		2.99	0.762	11.73
	Yes vs No	3.818	1.758	8.29		1.782	0.464	6.845		7.882	1.905	32.612

OR=Odds ratio; LCL=Lower 95% Confidence Interval; UCL=Upper 95% Confidence Interval

© 2021 Salter A et al. *JAMA Neurology.*

eTable 4. Multivariable logistic regression with the number of comorbid conditions.

Factor	Level	Hospitalization only				ICU and/or Ventilated				Death		
		OR	LCL	UCL		OR	LCL	UCL		OR	LCL	UCL
Age	(10 years)	1.298	1.108	1.52		1.177	0.915	1.512		1.619	1.11	2.362
Sex	Male vs Female	1.391	0.971	1.993		0.909	0.497	1.663		3.265	1.496	7.125
Race	Black/African American vs Non-Hispanic White	1.54	1.03	2.304		2.274	1.238	4.175		1.425	0.57	3.561
	Hispanic or Latinx vs Non- Hispanic White	0.797	0.429	1.479		1.737	0.756	3.989		0.604	0.111	3.285
	Other/Unknown vs Non- Hispanic White	0.854	0.343	2.126		0.813	0.177	3.743		1.621	0.219	11.989
Ambulation	Walk with Assistance vs Fully ambulatory	2.23	1.458	3.411		2.632	1.354	5.118		2.609	0.813	8.373
	Non-ambulatory vs Fully ambulatory	2.938	1.722	5.012		3.72	1.691	8.184		24.184	8.756	66.799
Smoking status	Current vs Never	1.144	0.551	2.379		-	-	-		2.823	0.709	11.233
	Past vs Never	1.085	0.734	1.603		1.1	0.611	1.98		1.238	0.551	2.784
	Unknown vs Never	1.201	0.573	2.52		1.487	0.492	4.496		1.833	0.363	9.266
Number of comorbid conditions	1 vs 0	1.344	0.89	2.029		0.883	0.439	1.774		1.726	0.54	5.52
	2 vs 0	1.936	1.213	3.089		1.442	0.683	3.044		7.318	2.341	22.877
	≥3 vs 0	2.353	1.309	4.229		4.354	1.985	9.552		18.501	5.791	59.107
Disease Modifying Therapy	Fumarates vs None	0.992	0.524	1.877		0.223	0.07	0.707		0.342	0.079	1.485

© 2021 Salter A et al. *JAMA Neurology.*

	S1PR vs None	0.651	0.266	1.595		0.76	0.276	2.096		0.911	0.166	5.002
	Glatiramer acetate vs None	1.056	0.467	2.387		-	-	-		0.601	0.104	3.48
	Injectables vs None	0.35	0.078	1.576		0.289	0.036	2.32		0.569	0.05	6.506
	Natalizumab vs None	0.688	0.318	1.486		0.08	0.01	0.631		0.898	0.209	3.866
	Ocrelizumab vs None	1.597	0.961	2.654		0.838	0.426	1.646		0.399	0.14	1.134
	Other vs None	1.189	0.452	3.13		0.496	0.105	2.348		0.642	0.114	3.616
	Rituximab vs None	4.253	1.977	9.153		1.672	0.537	5.204		2.018	0.371	10.973
	Teriflunomide vs None	0.825	0.34	2.004		0.281	0.061	1.301		0.437	0.071	2.701
Glucocorticoid use in the past 2 months	Unknown vs No	0.914	0.451	1.854		0.398	0.09	1.757		2.165	0.667	7.031
	Yes vs No	2.754	1.403	5.406		1.485	0.469	4.696		3.366	0.883	12.829

OR=Odds ratio; LCL=Lower 95% Confidence Interval; UCL=Upper 95% Confidence Interval

© 2021 Salter A et al. *JAMA Neurology.*

eFigure. North American distribution of MS patients reported in COViMS Registry, A) Canada, B) United States.

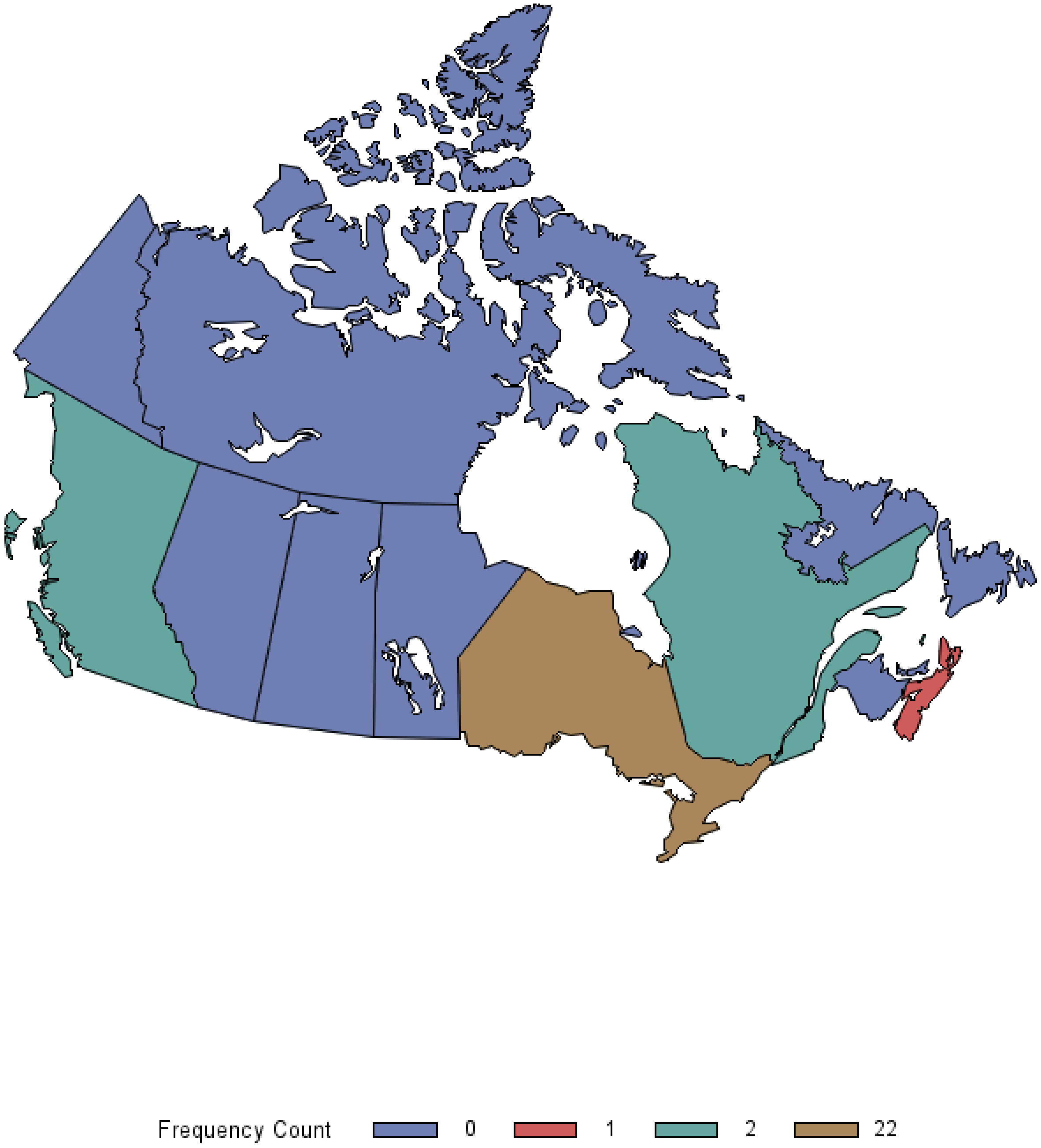

A.

© 2021 Salter A et al. *JAMA Neurology.*

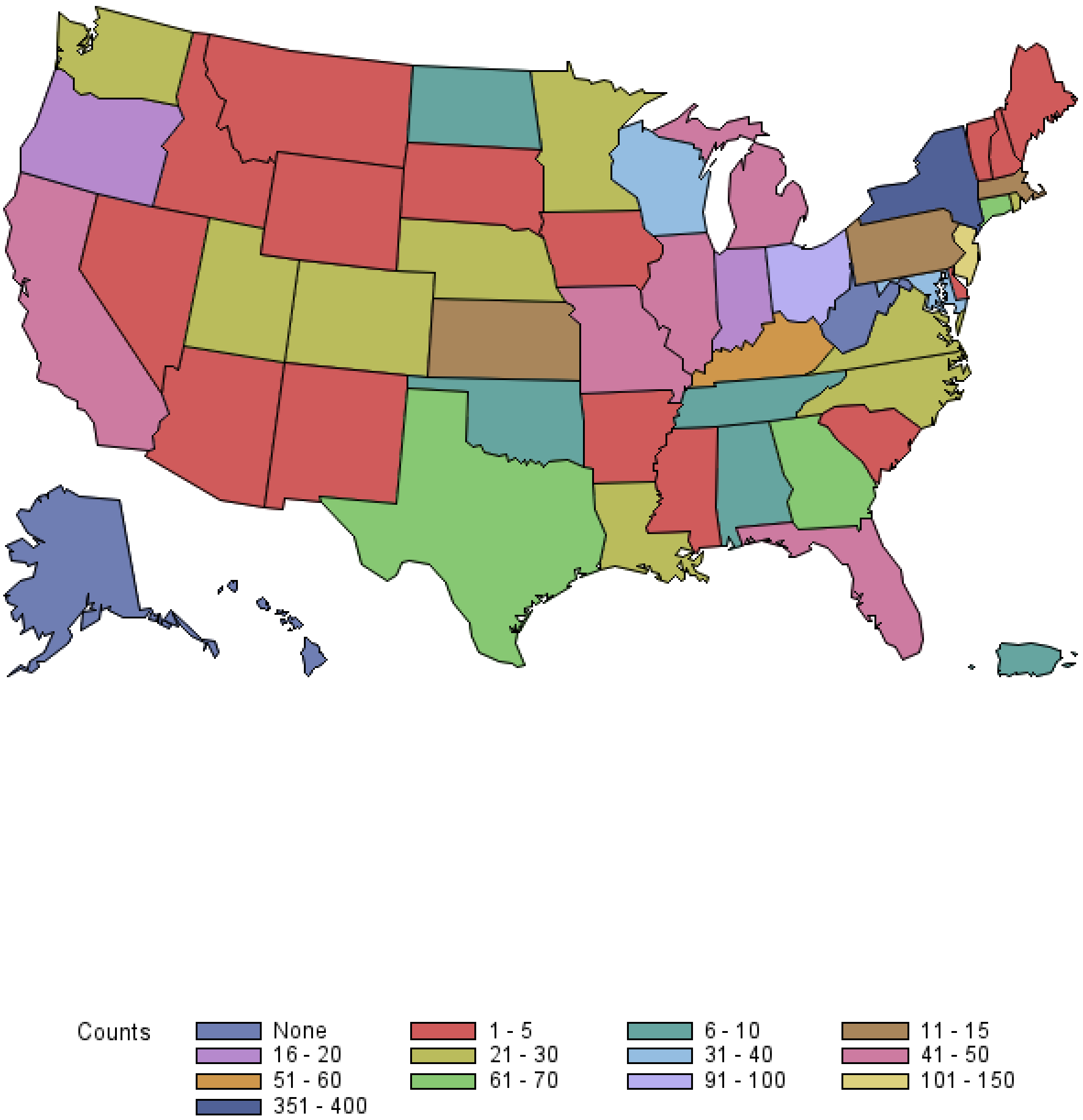

B.

Note: Mexico had 2 patients reported.

© 2021 Salter A et al. *JAMA Neurology.*
